# Association Between C‐Reactive Protein–Triglyceride Glucose Index and Adverse Cardiovascular Outcomes in Acute Coronary Syndrome Patients With Prior Coronary Artery Bypass Grafting

**DOI:** 10.1155/mi/7921309

**Published:** 2026-06-08

**Authors:** Xiaoteng Ma, Huijun Chu, Qiuxuan Li, Yuxiu Yang, Zhijian Wang

**Affiliations:** ^1^ Department of Cardiology, Beijing Anzhen Hospital, Capital Medical University, Beijing, China, ccmu.edu.cn; ^2^ Department of Anesthesia, Beijing Anzhen Hospital, Capital Medical University, Beijing, China, ccmu.edu.cn

**Keywords:** acute coronary syndrome, C-reactive protein–triglyceride glucose index, coronary artery bypass grafting, major adverse cardiovascular and cerebrovascular events, percutaneous coronary intervention

## Abstract

**Background:**

Patients with acute coronary syndrome (ACS) who have previously undergone coronary artery bypass grafting (CABG) represent a complex, high‐risk population characterized by a substantial burden of atherosclerosis and a marked propensity for recurrent ischemic events. The pathophysiological interplay between systemic inflammation and insulin resistance serves as a key mediator driving the progression of atherosclerosis and the destabilization of atherosclerotic plaques. However, the prognostic impact of their combined effect, quantified by a novel composite biomarker—the C‐reactive protein–triglyceride glucose index (CTI)—remains uncertain in this specific high‐risk population.

**Methods:**

We enrolled 1195 ACS patients with prior CABG who underwent percutaneous coronary intervention (PCI). The CTI was calculated as 0.412 × ln (high‐sensitivity C‐reactive protein [mg/L]) + ln (fasting triglycerides [mg/dL] × fasting blood glucose [mg/dL]/2). Patients were divided into three groups based on their CTI tertiles. The primary endpoint was defined as the occurrence of major adverse cardiovascular and cerebrovascular events (MACCE), which encompassed all‐cause death, nonfatal stroke, nonfatal myocardial infarction (MI), and unplanned revascularization.

**Results:**

Over a median follow‐up of 3 years, 366 patients experienced MACCE. The incidence of MACCE progressively increased across CTI tertiles (log‐rank *p*  < 0.001). In the multivariable Cox proportional hazards model adjusted for the GRACE (Global Registry of Acute Coronary Events) risk score and a comprehensive panel of clinical, procedural, and laboratory confounders, the highest CTI tertile remained an independent predictor of MACCE (adjusted hazard ratio [HR]: 3.864, 95% confidence interval [CI]: 2.710–5.511, *p*  < 0.001). When CTI was analyzed as a continuous variable, each unit increase was found to confer an 80.1% greater risk of MACCE (adjusted HR: 1.801, 95% CI: 1.556–2.085, *p*  < 0.001). This association remained consistent across all predefined subgroups. Adding CTI tertiles to the baseline model—which encompassed the GRACE risk score and other confounders—yielded a modest but statistically significant improvement in predictive performance (*C*‐statistic increased from 0.605 to 0.655, *p*  < 0.001; continuous net reclassification improvement [cNRI]: 0.740, *p* = 0.032; integrated discrimination improvement [IDI]: 0.145, *p* = 0.020).

**Conclusions:**

The CTI—a composite biomarker that captures both systemic inflammation and insulin resistance—emerged as a significant and independent predictor of long‐term MACCE in ACS patients with prior CABG who underwent PCI. Its integration into risk stratification models may improve prognostic assessment and potentially facilitate more personalized and intensive secondary prevention strategies.

## 1. Background

The clinical management of individuals with prior coronary artery bypass grafting (CABG) who present with acute coronary syndrome (ACS) represents one of the most significant challenges in contemporary cardiology [1, 2]. These patients experience significantly worse outcomes than those without a history of CABG [3–6]. They often exhibit diffuse, multivessel, and accelerated coronary artery disease affecting both native coronary arteries and bypass grafts [7]. Notably, culprit lesions in these patients are more frequently located in bypass grafts than in native coronary arteries [8, 9]. Saphenous vein grafts (SVGs), the most commonly used bypass conduits, are particularly prone to graft failure, which is marked by intimal hyperplasia, superimposed atherosclerosis, and thrombotic occlusion—processes that are often more aggressive and inflammatory than those observed in native vessel disease [10]. These pathophysiological alterations contribute to the elevated frequency of recurrent ischemic episodes and the considerable residual risk noted in this patient group, despite advances in coronary revascularization techniques and medical therapy [11]. When repeat revascularization is indicated, percutaneous coronary intervention (PCI) may be preferred over redo CABG [11, 12].

Two key pathophysiological mechanisms—systemic inflammation and insulin resistance—underlie the atherosclerosis progression and plaque instability [13–15]. Atherosclerosis is currently widely recognized as a chronic inflammatory disease involving the vascular wall [16, 17]. From the initial recruitment of monocytes to the endothelium, through atherosclerotic plaque formation, and up to the eventual rupture of vulnerable plaques, inflammatory cells and cytokines are involved at every stage of this process [16, 17]. C‐reactive protein (CRP), produced by hepatocytes primarily driven by interleukin‐6 (IL‐6) signaling, serves as a robust and clinically accessible biomarker of systemic inflammation [18, 19]. Very low levels of CRP that require a high‐sensitivity assay for detection are referred to as high‐sensitivity CRP (hsCRP), which is recommended as the preferred biomarker for assessing residual inflammatory risk [19]. Landmark studies, including the JUPITER (Justification for Use of statins in Prevention: an Intervention Trial Evaluating Rosuvastatin) [20] and CANTOS (Canakinumab Anti‐inflammatory Thrombosis Outcomes Study) trials [21], have firmly established that elevated levels of hsCRP are associated with a greater risk of subsequent cardiovascular events, independent of traditional risk factors. In the context of post‐CABG ACS, elevated hsCRP levels may reflect not only the instability of plaques in native coronary arteries but also active inflammatory processes within degenerating bypass grafts [22, 23].

Concurrently, insulin resistance—defined as impaired cellular responsiveness to insulin—has been established as a key determinant of cardiometabolic risk [13]. It represents the fundamental defect underlying type 2 diabetes and metabolic syndrome. Insulin resistance fosters a proatherogenic environment via several mechanisms, such as induction of dyslipidemia (increased triglyceride levels, decreased high‐density lipoprotein cholesterol [HDL‐C] levels, and an elevated number of small, dense low‐density lipoprotein [LDL] particles), promotion of endothelial dysfunction through impaired nitric oxide synthase activity, stimulation of vascular smooth muscle cell proliferation, and enhancement of proinflammatory signaling [13, 24]. The triglyceride–glucose (TyG) index has proven to be a simple, reliable, and cost‐effective proxy for insulin resistance [25, 26]. A growing body of evidence suggests that a high TyG index is closely linked to poor cardiovascular prognosis across diverse patient populations, often performing comparably to or even outperforming traditional measures such as the homeostasis model assessment of insulin resistance (HOMA‐IR) and the hyperinsulinemic–euglycemic clamp, which serves as the gold standard for evaluation [27, 28].

Importantly, systemic inflammation and insulin resistance are not isolated pathways but are deeply interconnected in a vicious cycle, a concept often referred to as “meta‐inflammation” [29]. Insulin resistance can stimulate proinflammatory signaling pathways, notably through the mechanism of nuclear factor kappa B (NF‐κB) activation. Conversely, insulin receptor substrate signaling can be impaired by inflammatory cytokines such as IL‐6 and tumor necrosis factor‐α (TNF‐α), which in turn aggravates insulin resistance [30, 31]. This synergistic relationship suggests that combined assessment may provide a more integrated and robust prognostic signal than either marker alone. Although hsCRP and the TyG index have been extensively studied separately, integrating them into a single composite index—the CRP–triglyceride glucose index (CTI)—to quantify this “meta‐inflammatory” burden is a novel concept. We hypothesized that CTI would provide superior prognostic information through the integration of metabolic and inflammatory pathways. Therefore, the present study sought to investigate the relationship between CTI and the risk of major adverse cardiovascular and cerebrovascular events (MACCE) in a well‐defined cohort of ACS patients with prior CABG.

## 2. Methods

### 2.1. Study Design and Population

**Figure 1:**
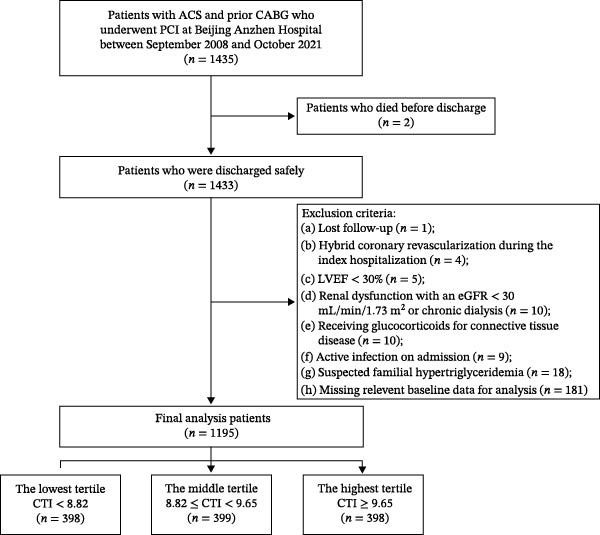
Flowchart illustrating the selection process of the study population. ACS, acute coronary syndrome; CABG, coronary artery bypass grafting; CTI, C‐reactive protein–triglyceride glucose index; eGFR, estimated glomerular filtration rate; LVEF, left ventricular ejection fraction; PCI, percutaneous coronary intervention.

This investigation was a retrospective analysis using data from a single‐center observational cohort, which was initially designed to explore the relationship between the PCI target vessel (native coronary arteries vs bypass grafts) and adverse cardiovascular outcomes (NCT05368597; https://www.clinicaltrials.gov/; registration date: 2022‐05‐06) [32]. We performed a screening of electronic medical records for all patients who had been admitted with a primary diagnosis of ACS between September 2008 and October 2021. From this population, we identified 1,435 consecutive adults (aged ≥18 years) who met the following inclusion criteria: (1) a confirmed diagnosis of ACS, which encompassed ST‐elevation myocardial infarction (STEMI), non‐STEMI (NSTEMI), or unstable angina (UA), as per current clinical guidelines [33, 34]; (2) a documented history of CABG; (3) treatment with PCI during the index hospitalization. To ensure clear interpretation of the results and minimize potential confounding, we excluded patients who died before discharge (two deaths occurred during hospitalization, both of which were considered to be related to the PCI procedure), were lost to follow‐up even after a minimum of four separate attempts to establish contact, had a left ventricular ejection fraction <30%, underwent hybrid coronary revascularization, had renal dysfunction defined as an estimated glomerular filtration rate (eGFR) <30 mL/min/1.73 m^2^ or required chronic dialysis, were receiving glucocorticoids for connective tissue disease, had active infection on admission, had suspected familial hypertriglyceridemia (plasma triglycerides ≥500 mg/dL), or lacked relevant baseline data for analysis. The final analysis included a cohort of 1195 patients (Figure 1). This study was performed in compliance with the ethical principles of the Declaration of Helsinki. The study protocol received clearance from the Institutional Review Board of Beijing Anzhen Hospital, Capital Medical University (Number 2025349x). Due to the study’s retrospective design, informed consent was not required.

### 2.2. Data Collection and Definitions

Baseline patient information—including demographics, clinical features, cardiovascular risk factors, and medical history—was systematically gathered from the electronic medical records of the hospital via a standardized data collection form. Physical examination findings, including heart rate and systolic blood pressure (SBP) at admission, were recorded. The GRACE (Global Registry of Acute Coronary Events) risk score—a tool that estimates the 6‐month postdischarge mortality risk—was calculated for each patient.

Blood samples were collected from each patient within 24 h of admission after a minimum 8‐h overnight fast. All laboratory assays were conducted at the central laboratory of the Beijing Anzhen Hospital. Standard laboratory techniques were used to measure serum creatinine, total cholesterol, HDL‐C, LDL cholesterol (LDL‐C), triglycerides, glycated hemoglobin A1c (HbA1c), and fasting blood glucose (FBG). hsCRP levels were quantified using an immunoturbidimetric assay. The eGFR was computed by means of the CKD‐EPI (Chronic Kidney Disease Epidemiology Collaboration) equation. Renal dysfunction was determined as an eGFR value below 60 mL/min/1.73 m^2^. Referring to previously published studies [35–38], CTI was calculated as 0.412 × ln (hsCRP [mg/L]) + ln (triglycerides [mg/dL] × FBG [mg/dL]/2). Using data from the INSCOC (Investigation on Nutrition Status and its Clinical Outcome of Common Cancers) study, Ruan et al. [35] determined that the weight coefficient of 0.412 in the CTI formula was derived through statistical analysis and model optimization, which made the CTI formula perform best in predicting the poor prognosis of patients.

Detailed information regarding the index PCI procedure was collected, including the vascular access site (radial/brachial, femoral, or both), the type of vessels treated (native coronary arteries only, graft vessels only, or both), and the specific native coronary arteries—right coronary artery, left anterior descending artery, left circumflex artery, and left main coronary artery (LMCA)—or graft vessels (SVGs and left internal mammary artery) involved. Target vessel revascularization was deemed successful if TIMI (thrombolysis in myocardial infarction [MI]) Grade 3 flow was established, residual stenosis was less than 20%, and no major in‐hospital procedural complications (e.g., no‐reflow phenomenon, distal embolization, or periprocedural MI) were observed. Information on medication use before admission and at hospital discharge was also documented.

### 2.3. Study Endpoints and Follow‐up

Trained personnel who were blinded to baseline characteristics collected information on adverse cardiovascular outcomes via telephone interviews with patients or their family members. The obtained data were subsequently verified through a thorough review of the corresponding medical records. The occurrence of MACCE during the follow‐up period served as the primary endpoint. The individual components of the primary composite endpoint, along with a key secondary composite endpoint consisting of all‐cause death, nonfatal stroke, or nonfatal MI, were included among the secondary endpoints. The last follow‐up assessment was performed in April 2022.

### 2.4. Statistical Analysis

Continuous variables with a normal distribution were presented as mean ± standard deviation and compared across CTI tertiles using one‐way analysis of variance (ANOVA). Continuous variables with a nonnormal distribution were presented as the median (interquartile range) and compared using the Kruskal–Wallis test. Categorical variables were presented as counts (percentages) and compared using either the chi‐square test or Fisher’s exact test, as appropriate. CTI was analyzed chiefly as a categorical variable—specifically, CTI tertiles using the lowest tertile as the reference—and, in addition, as a continuous variable (per 1‐unit increase) in sensitivity analyses. The optimal cutoff value of CTI for predicting MACCE was determined using receiver operating characteristic (ROC) curve analysis and Youden’s index (sensitivity + specificity − 1). The Kaplan–Meier method was applied to estimate the cumulative incidence of MACCE overtime, and the log‐rank test was used to compare the survival curves across CTI tertiles. Univariate and multivariable Cox proportional hazards regression models were utilized to evaluate the relationship between CTI and the risk of MACCE, with results expressed as hazard ratios (HRs) with corresponding 95% confidence intervals (CIs). Multivariable models were constructed by including covariates with a univariate significance level <0.20 and excluding variables that might introduce multicollinearity. The primary Cox model included CTI tertiles, the GRACE risk score, and other confounders (male sex, body mass index [BMI], diabetes, hypertension, renal dysfunction, prior PCI, previous stroke, chronic lung disease, LDL‐C, HDL‐C, HbA1c, years since CABG, whether the index PCI was the first PCI after CABG, PCI in native and/or graft vessels, intervention in the LMCA, intervention in SVGs, and successful target vessel revascularization). To evaluate the proportional hazards assumption within the Cox model, the Schoenfeld residual test was applied. For the primary Cox model including CTI tertiles, the global test yielded a nonsignificant *p* value (*p*  = 0.167), and no individual covariate demonstrated a significant violation of the proportional hazards assumption. To evaluate the robustness of our findings, we performed several sensitivity analyses: (1) including CTI as a continuous variable instead of CTI tertiles in the primary Cox model (global test *p* = 0.177 for the proportional hazards assumption); (2) replacing the GRACE risk score with its individual components (age, heart rate, SBP, etc.) in the primary Cox model (global test *p* = 0.367 for the proportional hazards assumption). Furthermore, the consistency of the association between CTI (analyzed as a continuous variable) and MACCE was examined via subgroup analyses across multiple predefined patient strata. These strata included age (<60 vs. ≥60 years), BMI (<24 vs. ≥24 kg/m^2^), sex (male vs. female), hypertension (yes vs. no), diabetes (yes vs. no), renal dysfunction (yes vs. no), prior PCI (yes vs. no), clinical presentation (UA vs. acute myocardial infarction [AMI]), and enrollment period (2008–2015 vs. 2016–2021). Interaction terms between CTI and subgroup variables were introduced into the Cox model to test for effect modification. To quantify the improvement in predictive performance when CTI was added to the baseline prediction model, the *C*‐statistic, continuous net reclassification improvement (cNRI), and integrated discrimination improvement (IDI) were derived. All statistical tests were two‐sided, with a *p* value <0.05 considered significant. SPSS (version 24.0, IBM Corp., Armonk, NY, USA) and R (version 4.1.0, R Foundation for Statistical Computing, Beijing, China) were employed for all statistical analyses.

## 3. Results

**Figure 2:**
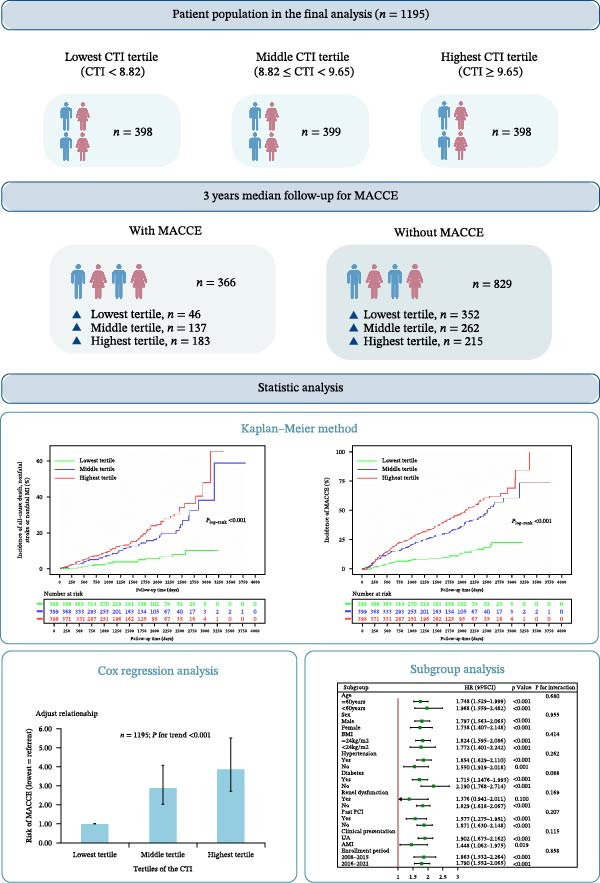
Schematic overview of the study design. The illustration was created using Adobe Illustrator. CTI, C‐reactive protein–triglyceride glucose index; MACCE, major adverse cardiovascular and cerebrovascular events.

The overall workflow and principal findings of the present study are illustrated in Figure 2. The baseline demographic and clinical profiles of the entire analyzed cohort (*n* = 1195) and their distribution across CTI tertiles are detailed in Table 1. The study population had a mean age of 64 ± 8 years, and the majority of patients were men, accounting for 75.8% of the cohort. As expected, CTI values increased significantly across tertiles (lowest: 8.25 ± 0.45; middle: 9.25 ± 0.24; highest: 10.17 ± 0.47; *p*  < 0.001). Patients in the highest CTI tertile exhibited a distinct cardiometabolic risk profile, with significantly higher TyG index and hsCRP levels. Notably, the proportion of male patients increased significantly, whereas that of female patients decreased, across increasing CTI tertiles. Correspondingly, mean CTI values were lower in male than in female patients (9.16 ± 0.88 vs. 9.42 ± 0.86, *p*  < 0.001). Furthermore, patients in the highest CTI tertile carried a heavier burden of traditional risk factors, notably a greater prevalence of both diabetes and hypertension, although the difference in the latter did not achieve statistical significance. Alterations in lipid metabolism were also prominent, with the highest CTI tertile exhibiting adverse lipid profiles characterized by higher total cholesterol, LDL‐C, and triglyceride levels, along with lower HDL‐C levels (all *p*  < 0.001). Glycemic control was poorer in the highest CTI tertile, as reflected by higher FBG and HbA1c levels, along with a higher utilization of hypoglycemic agents both at admission and on discharge. In addition, the highest CTI tertile corresponded to a heavier comorbidity burden, including renal dysfunction and a higher prevalence of prior PCI. Regarding clinical presentation, patients with higher CTI values were less likely to present with UA and more likely to present with NSTEMI or STEMI (*p*  < 0.001), consistent with a more unstable and severe ischemic phenotype. The GRACE risk score increased progressively across CTI tertiles (*p*  = 0.008). Procedural characteristics demonstrated a trend toward more frequent PCI in graft vessels among patients in higher CTI tertiles.

**Table 1:** Baseline characteristics of the study population according to CTI tertiles

Variable	All patients (*n* = 1195)	Lowest tertile (*n* = 398)	Middle tertile (*n* = 399)	Highest tertile (*n* = 398)	*p* value
CTI, mean ± SD	9.22 ± 0.88	8.25 ± 0.45	9.25 ± 0.24	10.17 ± 0.47	<0.001
TyG index, mean ± SD	9.04 ± 0.61	8.50 ± 0.36	9.06 ± 0.35	9.58 ± 0.54	<0.001
hsCRP (mg/L), median (IQR)	1.38 (0.69–3.38)	0.56 (0.32–0.95)	1.62 (0.91–2.74)	3.75 (1.81–9.47)	<0.001
Age (years), mean ± SD	64 ± 8	65 ± 8	65 ± 8	64 ± 8	0.067
Male sex, *n* (%)	906 (75.8)	331 (83.2)	291 (72.9)	284 (71.4)	<0.001
BMI (kg/m^2^), mean ± SD	26.2 ± 3.2	25.6 ± 3.1	26.3 ± 3.2	26.7 ± 3.2	<0.001
SBP at admission (mm Hg), mean ± SD	130 ± 17	130 ± 18	131 ± 18	130 ± 17	0.588
Heart rate at admission (bpm), mean ± SD	67 ± 10	65 ± 10	67 ± 10	69 ± 11	<0.001
Family history of CAD, *n* (%)	118 (9.9)	34 (8.5)	40 (10.0)	44 (11.1)	0.490
Current smoking, *n* (%)	287 (24.0)	85 (21.4)	99 (24.8)	103 (25.9)	0.296
Hypertension, *n* (%)	920 (77.0)	296 (74.4)	305 (76.4)	319 (80.2)	0.146
Diabetes, *n* (%)	679 (56.8)	169 (42.5)	215 (53.9)	295 (74.1)	<0.001
HF, *n* (%)	107 (9.0)	33 (8.3)	36 (9.0)	38 (9.5)	0.823
Renal dysfunction, *n* (%)	93 (7.8)	17 (4.3)	31 (7.8)	45 (11.3)	0.001
AF, *n* (%)	45 (3.8)	13 (3.3)	15 (3.8)	17 (4.3)	0.758
Previous MI, *n* (%)	588 (49.2)	184 (46.2)	198 (49.6)	206 (51.8)	0.290
Past PCI, *n* (%)	342 (28.6)	95 (23.9)	117 (29.3)	130 (32.7)	0.022
Previous stroke, *n* (%)	146 (12.2)	53 (13.3)	51 (12.8)	42 (10.6)	0.450
PAD, *n* (%)	118 (9.9)	36 (9.0)	38 (9.6)	44 (11.1)	0.611
Chronic lung disease, *n* (%)	56 (4.7)	19 (4.8)	18 (4.5)	19 (4.8)	0.980
Clinical presentation					<0.001
UA, *n* (%)	1,009 (84.4)	370 (93.0)	341 (85.5)	298 (74.9)	
NSTEMI, *n* (%)	149 (12.5)	26 (6.5)	46 (11.5)	77 (19.3)	
STEMI, *n* (%)	37 (3.1)	2 (0.5)	12 (3.0)	23 (5.8)	
GRACE risk score, mean ± SD	90 ± 21	87 ± 20	90 ± 21	92 ± 23	0.008
Laboratory measurements (fasting state)					
SCr (umol/L), mean±SD	77.2 ± 18.8	75.4 ± 15.2	76.9 ± 18.9	79.4 ± 21.6	0.011
TC (mg/dL), mean±SD	153.3 ± 39.0	140.4 ± 35.2	154.5 ± 39.5	164.9 ± 38.3	<0.001
LDL‐C (mg/dL), mean±SD	90.5 ± 33.8	81.2 ± 31.8	92.0 ± 34.0	98.3 ± 33.5	<0.001
HDL‐C (mg/dL), mean±SD	38.4 ± 8.9	41.6 ± 9.0	38.1 ± 8.4	35.7 ± 8.2	<0.001
Triglycerides (mg/dL), median (IQR)	136.4 (100.1−186.0)	96.5 (77.1−119.6)	149.7 (117.8−179.8)	184.7 (139.9−251.8)	<0.001
FBG (mg/dL), median (IQR)	112.6 (96.8−144.2)	98.7 (89.8−114.5)	110.8 (97.8−132.3)	144.0 (114.3−204.2)	<0.001
HbA1c (%), median (IQR)	6.4 (5.9−7.4)	6.1 (5.7−6.8)	6.4 (5.9−7.1)	7.1 (6.1−8.2)	<0.001
Years since CABG, median (IQR)	6.0 (3.0−10.0)	5.0 (2.0−10.0)	6.0 (3.0−10.0)	6.0 (3.0−10.0)	0.014
The index PCI as the first PCI after CABG, *n* (%)	1095 (91.6)	374 (94.0)	358 (89.7)	363 (91.2)	0.090
Percutaneous entry					0.258
Radial/brachial only, *n* (%)	603 (50.5)	212 (53.3)	196 (49.1)	195 (49.0)	
Femoral only, *n* (%)	547 (45.8)	174 (43.7)	191 (47.9)	182 (45.7)	
Radial/brachial + femoral, *n* (%)	45 (3.8)	12 (3.0)	12 (3.0)	21 (5.3)	
Procedure results					
PCI in native and/or graft vessels					0.062
PCI in only native vessels, *n* (%)	1013 (84.8)	349 (87.7)	336 (84.2)	328 (82.4)	
PCI in only graft vessels, *n* (%)	122 (10.2)	27 (6.8)	43 (10.8)	52 (13.1)	
PCI in both native and graft vessels, *n* (%)	60 (5.0)	22 (5.5)	20 (5.0)	18 (4.5)	
Native vessel intervened, *n* (%)					
LMCA	144 (12.1)	60 (15.1)	44 (11.0)	40 (10.1)	0.070
LAD	292 (24.4)	112 (28.1)	95 (23.8)	85 (21.4)	0.079
LCX	412 (34.5)	130 (32.7)	143 (35.8)	139 (34.9)	0.624
RCA	572 (47.9)	193 (48.5)	186 (46.6)	193 (48.5)	0.829
Graft vessel intervened, *n* (%)					
LIMA	4 (0.3)	3 (0.8)	0 (0)	1 (0.3)	0.135
SVG	178 (14.9)	46 (11.6)	63 (15.8)	69 (17.3)	0.060
Target vessel revascularization successful, *n* (%)	1156 (96.7)	389 (97.7)	385 (96.5)	382 (96.0)	0.356
Use of hypoglycemic agents before admission, *n* (%)	517 (43.3)	135 (33.9)	150 (37.6)	232 (58.3)	<0.001
Use of medications at discharge					
Aspirin, *n* (%)	1185 (99.2)	396 (99.5)	393 (98.5)	396 (99.5)	0.325
P2Y12 inhibitors, *n* (%)	1185 (99.2)	394 (99.0)	393 (98.5)	398 (100.0)	0.043
Oral anticoagulants, *n* (%)	37 (3.1)	19 (4.8)	12 (3.0)	6 (1.5)	0.029
Statins, *n* (%)	1184 (99.1)	396 (99.5)	393 (98.5)	395 (99.2)	0.410
β‐Blockers, n (%)	1009 (84.4)	335 (84.2)	333 (83.5)	341 (85.7)	0.677
ACEI/ARB/ARNIs, *n* (%)	630 (52.7)	192 (48.2)	207 (51.9)	231 (58.0)	0.020
Insulin, *n* (%)	251 (21.0)	73 (18.3)	62 (15.5)	116 (29.1)	<0.001
Oral hypoglycemic agents, *n* (%)	405 (33.9)	102 (25.6)	126 (31.6)	177 (44.5)	<0.001

Abbreviations: ACEI/ARB/ARNIs, angiotensin converting enzyme inhibitors/angiotensin receptor blockers/angiotensin receptor‐neprilysin inhibitors; AF, atrial fibrillation/flutter; BMI, body mass index; CABG, coronary artery bypass grafting; CAD, coronary artery disease; CTI, C‐reactive protein–triglyceride glucose index; FBG, fasting blood glucose; HbA1c, glycated hemoglobin A1c; HDL‐C, high‐density lipoprotein cholesterol; HF, heart failure; hsCRP, high‐sensitivity C‐reactive protein; LAD, left anterior descending artery; LCX, left circumflex artery; LDL‐C, low‐density lipoprotein cholesterol; LIMA, left internal mammary artery; LMCA, left main coronary artery; LVEF, left ventricular ejection fraction; MI, myocardial infarction; NSTEMI, non‐ST‐segment elevation myocardial infarction; PAD, peripheral artery disease; PCI, percutaneous coronary intervention; RCA, right coronary artery; SBP, systolic blood pressure; SCr, serum creatinine; STEMI, ST‐segment elevation myocardial infarction; SVG, saphenous vein graft; TC, total cholesterol; TyG, triglyceride–glucose; UA, unstable angina.

Over a median follow‐up duration of 3 years, 366 patients (30.6%) experienced MACCE. The rate of MACCE rose across CTI tertiles, ranging from 11.6% in the lowest tertile to 46.0% in the highest tertile. The corresponding event counts and incidence rates of individual MACCE components across CTI tertiles are shown in Supporting Information 1: Table S1. A comparative analysis of baseline demographic and clinical profiles between patients with and without MACCE is shown in Table 2. Patients who developed MACCE had significantly higher baseline CTI values than those who did not (9.65 ± 0.71 vs. 9.03 ± 0.88, *p*  < 0.001). The MACCE group exhibited more pronounced metabolic and inflammatory dysregulation, characterized by a higher TyG index and hsCRP levels and less favorable lipid and glycemic parameters. Clinically, these patients had higher SBP and heart rate at admission and a greater prevalence of hypertension, renal dysfunction, and prior PCI. Procedural characteristics also differed, with the MACCE group demonstrating a decreased rate of successful target vessel revascularization and an increased tendency toward PCI in graft vessels.

**Table 2:** Baseline characteristics of the study population grouped by MACCE

Variable	Patients without MACCE (*n* = 829)	Patients with MACCE (*n* = 366)	*p* Value
CTI, mean ± SD	9.03 ± 0.88	9.65 ± 0.71	<0.001
Lowest tertile, *n* (%)	352 (42.5)	46 (12.6)	—
Middle tertile, *n* (%)	262 (31.6)	137 (37.4)	—
Highest tertile, *n* (%)	215 (25.9)	183 (50.0)	—
TyG index, mean ± SD	8.94 ± 0.61	9.27 ± 0.56	<0.001
hsCRP (mg/L), median (IQR)	1.08 (0.50−2.83)	2.12 (1.18−4.49)	<0.001
Age (years), mean±SD	64 ± 8	65 ± 8	0.401
Male sex, *n* (%)	635 (76.6)	271 (74.0)	0.342
BMI (kg/m^2^), mean±SD	26.1 ± 3.1	26.5 ± 3.4	0.024
SBP at admission (mm Hg), mean ± SD	129 ± 17	132 ± 19	0.034
Heart rate at admission (bpm), mean ± SD	67 ± 10	68 ± 10	0.010
Family history of CAD, *n* (%)	71 (8.6)	47 (12.8)	0.022
Current smoking, *n* (%)	192 (23.2)	95 (26.0)	0.297
Hypertension, *n* (%)	624 (75.3)	296 (80.9)	0.034
Diabetes, *n* (%)	462 (55.7)	217 (59.3)	0.252
HF, *n* (%)	75 (9.0)	32 (8.7)	0.865
Renal dysfunction, *n* (%)	51 (6.2)	42 (11.5)	0.002
AF, *n* (%)	31 (3.7)	14 (3.8)	0.943
Previous MI, *n* (%)	400 (48.3)	188 (51.4)	0.321
Past PCI, *n* (%)	222 (26.8)	120 (32.8)	0.034
Previous stroke, *n* (%)	98 (11.8)	48 (13.1)	0.529
PAD, *n* (%)	78 (9.4)	40 (10.9)	0.417
Chronic lung disease, *n* (%)	43 (5.2)	13 (3.6)	0.218
Clinical presentation			0.389
UA, *n* (%)	705 (85.0)	304 (83.1)	
NSTEMI, *n* (%)	102 (12.3)	47 (12.8)	
STEMI, *n* (%)	22 (2.7)	15 (4.1)	
GRACE risk score, mean ± SD	89 ± 21	91 ± 21	0.152
Laboratory measurements (fasting state)			
SCr (umol/L), mean ± SD	76.3 ± 17.0	79.3 ± 22.3	0.021
TC (mg/dL), mean ± SD	150.4 ± 37.3	159.8 ± 42.0	<0.001
LDL‐C (mg/dL), mean ± SD	87.9 ± 32.4	96.4 ± 36.1	<0.001
HDL‐C (mg/dL), mean ± SD	39.1 ± 8.7	36.9 ± 9.0	<0.001
Triglycerides (mg/dL), median (IQR)	126.7 (93.9−177.1)	159.0 (120.5−201.5)	<0.001
FBG (mg/dL), median (IQR)	106.9 (93.8−137.4)	123.3 (106.0−161.8)	<0.001
HbA1c (%), median (IQR)	6.4 (5.8−7.3)	6.5 (5.9−7.5)	0.019
Years since CABG, median (IQR)	6.0 (2.5−10.0)	6.0 (3.0−10.0)	0.243
The index PCI as the first PCI after CABG, *n* (%)	770 (92.9)	325 (88.8)	0.019
Percutaneous entry			0.064
Radial/brachial only, *n* (%)	437 (52.7)	166 (45.4)	
Femoral only, *n* (%)	362 (43.7)	185 (50.5)	
Radial/brachial + femoral, *n* (%)	30 (3.6)	15 (4.1)	
Procedure results			
PCI in native and/or graft vessels			0.024
PCI in only native vessels, *n* (%)	712 (85.9)	301 (82.2)	
PCI in only graft vessels, *n* (%)	72 (8.7)	50 (13.7)	
PCI in both native and graft vessels, *n* (%)	45 (5.4)	15 (4.1)	
Native vessel intervened, *n* (%)			
LMCA	109 (13.1)	35 (9.6)	0.079
LAD	209 (25.2)	83 (22.7)	0.347
LCX	276 (33.3)	136 (37.2)	0.195
RCA	403 (48.6)	169 (46.2)	0.437
Graft vessel intervened, *n* (%)			
LIMA	3 (0.4)	1 (0.3)	1.000
SVG	114 (13.8)	64 (17.5)	0.095
Target vessel revascularization successful, *n* (%)	809 (97.6)	347 (94.8)	0.013
Use of hypoglycemic agents before admission, *n* (%)	350 (42.2)	167 (45.6)	0.273
Use of medications at discharge			
Aspirin, *n* (%)	822 (99.2)	363 (99.2)	1.000
P2Y12 inhibitors, *n* (%)	822 (99.2)	363 (99.2)	1.000
Oral anticoagulants, *n* (%)	30 (3.6)	7 (1.9)	0.117
Statins, *n* (%)	821 (99.0)	363 (99.2)	1.000
β‐Blockers, *n* (%)	703 (84.8)	306 (83.6)	0.600
ACEI/ARB/ARNIs, *n* (%)	422 (50.9)	208 (56.8)	0.059
Insulin, *n* (%)	173 (20.9)	78 (21.3)	0.862
Oral hypoglycemic agents, *n* (%)	275 (33.2)	130 (35.5)	0.430

Abbreviation: MACCE, major adverse cardiovascular and cerebrovascular event.

**Figure 3:**
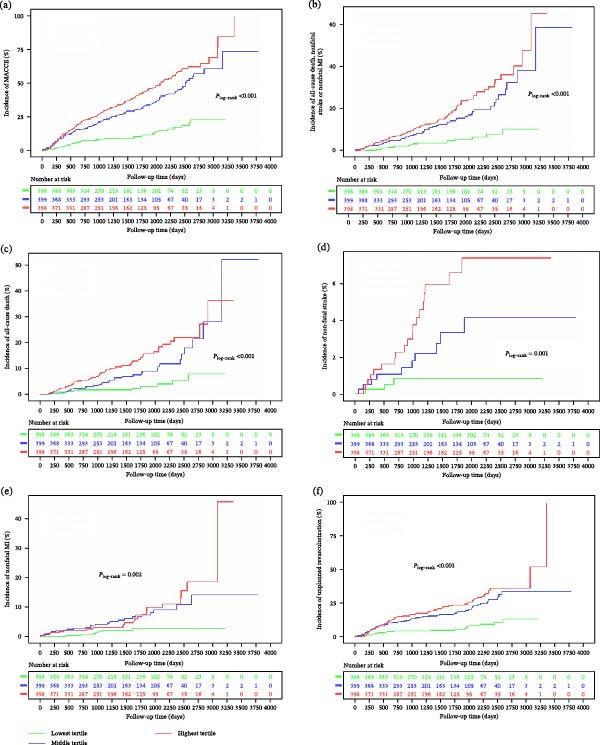
Kaplan–Meier curves showing the incidence of MACCE (a), the key secondary endpoint (b), all‐cause death (c), nonfatal stroke (d), nonfatal MI (e), and unplanned revascularization (f) among the three study groups stratified by CTI tertiles. The key secondary endpoint was defined as a composite of all‐cause death, nonfatal stroke, and nonfatal MI. CTI, C‐reactive protein–triglyceride glucose index; MACCE, major adverse cardiovascular and cerebrovascular events; MI, myocardial infarction.

Kaplan–Meier curves demonstrated a marked, stepwise escalation in MACCE risk with rising CTI tertiles (log‐rank *p*  < 0.001; Figure 3a). The same trend was also seen for the key secondary endpoint (log‐rank *p*  < 0.001; Figure 3b) and for individual MACCE components, including all‐cause death (log‐rank *p*  < 0.001; Figure 3c), nonfatal stroke (log‐rank *p* = 0.002; Figure 3d), nonfatal MI (log‐rank *p* = 0.002; Figure 3e), and unplanned revascularization (log‐rank *p*  < 0.001; Figure 3f).

**Figure 4:**
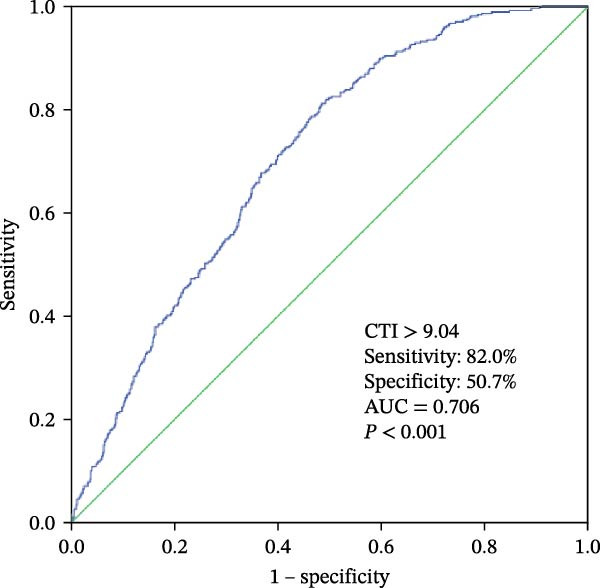
ROC analysis demonstrating the cutoff value of CTI for predicting MACCE. CTI, C‐reactive protein–triglyceride glucose index; MACCE, major adverse cardiovascular and cerebrovascular events; ROC, receiver operating characteristic.

The results of univariate and multivariable Cox proportional hazards analyses for predicting MACCE are summarized in Table 3 and Supporting Information 2–4: Tables S2–S4. In univariate Cox regression analysis, CTI was a robust predictor of MACCE. Using the lowest tertile as the reference, the middle and highest tertiles had HRs of 3.173 (95% CI: 2.271–4.432) and 4.308 (95% CI: 3.118–5.953), respectively (both *p*  < 0.001; Table 3). When analyzed as a continuous variable, CTI corresponded to an HR of 1.779 (95% CI: 1.602–2.019; *p*  < 0.001; Supporting Information 2: Table S2). After multivariable adjustment for the GRACE risk score and other confounders, the highest CTI tertile was found to remain independently predictive of MACCE (adjusted HR: 3.864, 95% CI: 2.710–5.511, *p*  < 0.001), and the middle tertile also showed a significant persistent association with increased risk (adjusted HR: 2.883, 95% CI: 2.039–4.078, *p*  < 0.001; Table 3). Each unit increase in CTI—when treated as a continuous variable—was associated with an 80.1% higher risk of MACCE (adjusted HR: 1.801, 95% CI: 1.556–2.085, *p*  < 0.001; Supporting Information 2: Table S2). In ROC analysis, a CTI cutoff value of 9.04 yielded a sensitivity of 82.0% and a specificity of 50.7% for predicting the primary endpoint (area under the curve [AUC] = 0.706, 95% CI: 0.676–0.736, *p*  < 0.001; Figure 4). Patients with CTI ≥9.04 exhibited a greater risk of MACCE compared to those with CTI <9.04 (adjusted HR: 2.922; 95% CI: 2.196–3.888; *p*  < 0.001). The robustness of the association between CTI and MACCE was further confirmed in sensitivity analyses in which the GRACE risk score was replaced with its core components (age, heart rate, SBP, and previous MI), with CTI retaining its strong and independent predictive value (Supporting Information 3–4: Tables S3 and S4). After adjusting for the GRACE risk score and other confounders in the multivariable Cox proportional hazards model, each unit increase in hsCRP and the TyG index was associated with an adjusted HR of 1.012 (95% CI: 0.996–1.028; *p* = 0.135) and 1.657 (95% CI: 1.369–2.005; *p* < 0.001), respectively.

**Table 3:** Multivariable Cox proportional hazards model including CTI as a categorical variable (CTI tertiles), GRACE risk score, and other confounding factors for predicting MACCE.

Variables	Univariate analysis		Multivariate analysis	
	HR (95% CI)	*p* value	HR (95% CI)	*p* value
CTI		<0.001		<0.001
Lowest tertile	ref		ref	
Middle tertile	3.173 (2.271−4.432)	<0.001	2.883 (2.039−4.078)	<0.001
Highest tertile	4.308 (3.118−5.953)	<0.001	3.864 (2.710−5.511)	<0.001
GRACE risk score	1.007 (1.002−1.011)	0.006	1.002 (0.997−1.008)	0.359
Male sex	0.850 (0.673−1.074)	0.173	0.991 (0.713−1.165)	0.457
BMI	1.032 (0.999−1.067)	0.054	1.005 (0.972−1.040)	0.762
Hypertension	1.292 (0.995−1.677)	0.054	1.151 (0.879−1.506)	0.308
Diabetes	1.173 (0.952−1.446)	0.134	0.822 (0.626−1.080)	0.160
Renal dysfunction	1.530 (1.109−2.112)	0.010	1.214 (0.860−1.713)	0.271
Past PCI	1.300 (1.045−1.617)	0.019	1.205 (0.930−1.563)	0.158
Previous stroke	1.272 (0.938−1.724)	0.121	1.295 (0.948−1.769)	0.105
Chronic lung disease	0.651 (0.374−1.134)	0.130	0.669 (0.383−1.167)	0.156
LDL‐C	1.005 (1.002−1.008)	<0.001	1.003 (1.000−1.006)	0.058
HDL‐C	0.983 (0.971−0.995)	0.006	0.998 (0.985−1.012)	0.793
HbA1c	1.101 (1.025−1.183)	0.009	1.043 (0.948−1.147)	0.391
Years since CABG	1.034 (1.011−1.057)	0.003	1.021 (0.995−1.047)	0.113
The index PCI as the first PCI after CABG	0.706 (0.510−0.979)	0.037	0.894 (0.595−1.344)	0.590
PCI in native and/or graft vessels		0.037		0.154
PCI in only native vessels	ref		ref	
PCI in only graft vessels	1.436 (1.064−1.938)	0.018	3.773 (0.517−27.522)	0.190
PCI in both native and graft vessels	0.807 (0.480−1.357)	0.418	2.429 (0.312−18.924)	0.397
Native vessel intervened: LMCA	0.681 (0.481−0.966)	0.031	0.842 (0.590−1.202)	0.344
Graft vessel intervened: SVG	1.215 (0.928−1.591)	0.157	0.296 (0.040−2.193)	0.234
Target vessel revascularization successful	0.577 (0.364−0.916)	0.020	0.617 (0.385−0.988)	0.044

Abbreviations: 95% CI, 95% confidence interval; HR, hazard ratio.

**Figure 5:**
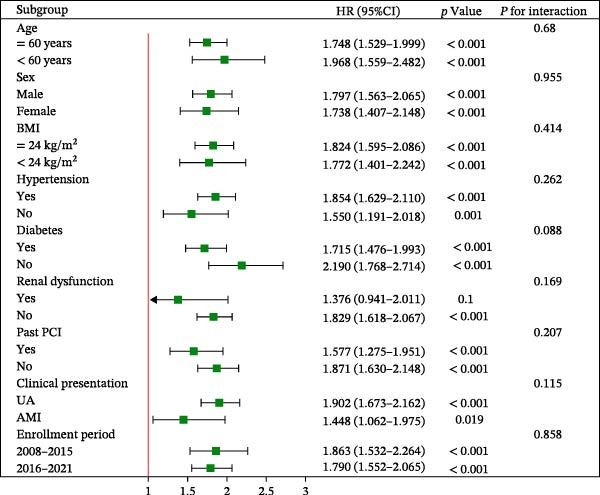
Subgroup analyses evaluating CTI as a continuous variable for predicting MACCE. HRs were calculated per 1‐unit increase in CTI. AMI, acute myocardial infarction; BMI, body mass index; CI, confidence interval; CTI, C‐reactive protein–triglyceride glucose index; HR, hazard ratio; MACCE, major adverse cardiovascular and cerebrovascular events; PCI, percutaneous coronary intervention; UA, unstable angina.

Subgroup analyses confirmed the consistent prognostic value of CTI (as a continuous variable) across predefined subgroups, including those stratified by age, sex, BMI, diabetes, hypertension, renal dysfunction, prior PCI, clinical presentation (UA vs. AMI), and enrollment period (2008–2015 vs. 2016–2021; Figure 5). No significant interactions were observed, supporting the robustness of the association. Notably, although the HR point estimate was numerically higher in non‐diabetic patients (HR: 2.190) than in patients with diabetes (HR: 1.715), the interaction term did not reach statistical significance (*p* = 0.088).

Adding of CTI tertiles to the baseline model—which encompassed the GRACE risk score and other confounders—yielded a modest but statistically significant improvement in predictive performance (*C*‐statistic increased from 0.605 [95% CI: 0.573–0.637] to 0.655 [95% CI: 0.626–0.683], *p*  < 0.001; cNRI: 0.740 [95% CI: 0.254–1.135], *p* = 0.032; IDI: 0.145 [95% CI: 0.039–0.206], *p* = 0.020). The addition of TyG index tertiles also improved the predictive performance of the same baseline model for MACCE (C‐statistic increased from 0.605 [95% CI: 0.573–0.637] to 0.641 [95% CI: 0.611–0.670], *p*  < 0.001; cNRI: 0.673 [95% CI: 0.130–1.075], *p* = 0.036; IDI: 0.173 [95% CI: 0.068–0.267], *p* = 0.010). Compared with the baseline model including only the GRACE risk score, *C*‐statistics increased significantly with the addition of CTI and TyG index tertiles, from 0.541 (95% CI: 0.507–0.575) to 0.640 (95% CI: 0.611–0.668) and 0.620 (95% CI: 0.591–0.649), respectively (all *p*  < 0.001).

The CTI formula incorporates hsCRP and the TyG index. We compared the predictive ability of the CTI, TyG index, and hsCRP after adding each biomarker to the baseline model—including the GRACE risk score and other confounders—for MACCE. The *C*‐statistics for CTI, TyG index, and hsCRP were 0.663 (95% CI: 0.635–0.690), 0.643 (95% CI: 0.613–0.672), and 0.606 (95% CI: 0.574–0.638), respectively. Pairwise comparisons of *C*‐statistics demonstrated that CTI had the highest discriminative ability (vs. TyG index, *p* = 0.003; vs. hsCRP, *p*  < 0.001), followed by the TyG index (vs. hsCRP, *p*  < 0.001).

## 4. Discussion

In this large contemporary cohort of ACS patients with a history of CABG who underwent PCI, we demonstrated for the first time that CTI—a newly developed composite index that integrates systemic inflammation and insulin resistance—was a strong and independent predictor of long‐term adverse cardiovascular outcomes. The main findings of this study are outlined as follows: First, a strong, graded relationship was observed between increasing CTI tertiles and the risk of MACCE, with cumulative incidence rising markedly across tertiles. Second, this relationship maintained its robustness even after extensive multivariable adjustment for a wide range of confounders, including the GRACE risk score and key clinical, procedural, and laboratory parameters. When analyzed as a continuous variable, each unit increase in CTI was associated with an 80.1% higher risk of MACCE, underscoring its linear relationship with adverse outcomes. Third, we identified the optimal cutoff value of the CTI for predicting MACCE. Compared with patients with CTI <9.04, those with CTI ≥9.04 had a significantly higher risk of MACCE. Fourth, across all predefined patient subgroups, the predictive significance of CTI remained consistent, supporting its generalizability in this complex population. Fifth, female ACS patients with prior CABG had higher baseline CTI values than male patients, suggesting a greater cardiometabolic and inflammatory risk profile in women. Higher CTI values have been shown to be significantly associated with a greater risk of adverse cardiovascular events. Consistent with this observation, female patients had a slightly higher incidence of MACCE (32.9%) compared with male patients (29.9%) during follow‐up. Notably, although CTI values were higher in female patients, subgroup analyses supported that CTI had comparable prognostic significance across both women and men. Finally, adding CTI to the baseline model—which encompassed the GRACE risk score and other confounders—enhanced both risk discrimination and reclassification, as demonstrated by an increment in the *C*‐statistic as well as statistically significant cNRI and IDI. Although the absolute increase in the *C*‐statistic was modest, the significant improvement in reclassification indices (cNRI and IDI) suggests that CTI contributes incremental prognostic information, exceeding what is available from traditional risk factors. Notably, CTI showed a stronger predictive performance for MACCE compared to either the TyG index or hsCRP.

The biological plausibility of our observations rests on the well‐established pathophysiological interplay between systemic inflammation and insulin resistance [39, 40]—a synergy often referred to as “meta‐inflammation” [29]—which may be amplified in the post‐CABG ACS setting. Atherosclerosis is fundamentally an inflammatory disease, and hsCRP, as a downstream marker of IL‐6–mediated inflammation [18], serves as a clinically accessible indicator of systemic inflammatory activity [19]. In the specific context of post‐CABG ACS, elevated hsCRP levels may reflect not only instability of native coronary artery plaques but also active inflammatory processes within degenerating SVGs, which are prone to aggressive inflammatory atherogenesis characterized by diffuse, lipid‐rich plaques with dense inflammatory infiltrates, large necrotic cores, and thin fibrous caps, rendering them highly vulnerable to rupture and thrombosis [22, 23]. Concurrently, insulin resistance—reliably quantifiable by the TyG index [25]—promotes a proatherogenic lipid profile (an elevation in triglycerides, a reduction in HDL‐C, and an increased number of small, dense LDL particles) and contributes to endothelial dysfunction by impairing the phosphoinositide 3‐kinase/Akt signaling pathway, resulting in reduced nitric oxide production and bioavailability and thereby fostering a proatherogenic milieu [13, 24]. In addition, it has been demonstrated that insulin resistance decreases the survival of vascular smooth muscle cells and contributes to the vulnerability of atherosclerotic plaques [41]. Importantly, insulin resistance in adipose tissue promotes a chronic low‐grade inflammatory state by enhancing the secretion of proinflammatory adipocytokines (e.g., TNF‐α, IL‐6, and monocyte chemoattractant protein‐1) while reducing the release of the anti‐inflammatory adipokine adiponectin. These cytokines enter the systemic circulation, activating endothelial cells, promoting monocyte recruitment, and inducing the liver to generate acute‐phase reactants such as hsCRP. Together, these processes create a vicious feed‐forward cycle: insulin resistance can trigger proinflammatory pathways like NF‐κB, whereas inflammatory cytokines—such as TNF‐α and IL‐6—can disrupt insulin signaling at the receptor level, thereby exacerbating insulin resistance [39, 40]. CTI, by mathematically combining the TyG index (a sensitive marker of insulin resistance) with hsCRP (a direct indicator of systemic inflammation), may provide an integrated quantification of this reciprocal interaction [37, 42]. The finding that CTI outperformed both the TyG index and hsCRP in predicting MACCE suggests that the combined assessment of these two pathways offers a more comprehensive representation of the underlying pathogenic mechanisms in this high‐risk cohort than either marker alone.

Our results further extend the existing evidence regarding the prognostic significance of systemic inflammation and insulin resistance for adverse cardiovascular events across diverse patient populations. Although the independent predictive significance of hsCRP for prognosis in primary and secondary prevention settings is well established, as supported by clinical trials including JUPITER [20] and CANTOS [21], and the TyG index is now widely viewed as a dependable surrogate indicator of insulin resistance and cardiovascular risk [28, 43], integrating these two biomarkers into a single index for risk prediction represents a novel conceptual advance. CTI has been shown to be a predictor of cardiovascular morbidity and mortality across diverse populations, including the general population and multiple patient groups. A study drawing on the China Health and Retirement Longitudinal Study database, which enrolled 8,084 individuals of middle to older age, demonstrated that higher CTI corresponded to an elevated risk of incident cardiovascular disease, defined as physician‐diagnosed heart disease or stroke, or current use of medication for heart disease or stroke [44]. An analysis of 10,718 individuals across Stages 0–3 of cardiovascular‐kidney‐metabolic syndrome in the National Health and Nutrition Examination Survey from 1999 to 2010 reported that higher CTI was independently associated with elevated risks of all‐cause death and cardiovascular death—where cardiovascular death was defined as mortality from cardiovascular or cerebrovascular causes [45]. A retrospective analysis drawing on the Medical Information Mart for Intensive Care IV database, which included 2428 critically ill patients between 2008 and 2022, showed that elevated CTI levels were significantly and independently associated with increased 30‐day and 1‐year all‐cause mortality and longer hospital stay [38]. The study by Gao et al. [37], which included 2383 PCI–treated patients—more than half of whom presented with ACS and approximately 20% of whom had previously undergone coronary revascularization (the proportion with prior CABG was not reported)—found that higher CTI independently and significantly correlated with an elevated risk of a composite endpoint comprising cardiovascular death, nonfatal ischemic stroke, nonfatal MI, and repeat coronary revascularization. Furthermore, CTI was more effective in identifying high‐risk patients than other markers of inflammation or insulin resistance, including the neutrophil‐to‐lymphocyte ratio, the TyG index, and the triglyceride‐to‐HDL‐C ratio [37]. To date, however, no studies have specifically examined the prognostic significance of CTI among ACS patients with prior CABG who underwent PCI. These patients have significantly worse outcomes—such as increased rates of periprocedural complications, recurrent ischemic events, and long‐term death—than those without prior CABG [46, 47]. Our investigation fills this critical gap in the literature by examining the prognostic role of CTI in this particularly vulnerable population. A study by Dong et al. [48] reported an independent association between the TyG index and MACCE in ACS patients with prior CABG who underwent PCI. Notably, our results indicate that the CTI outperforms the TyG index in predicting adverse cardiovascular outcomes. The robustness of our findings across multiple sensitivity and subgroup analyses further supports the potential clinical utility of CTI. Importantly, the association of CTI with MACCE persisted in both patients with and without diabetes, suggesting that CTI may reflect a broader pathophysiological state beyond conventional glucose metabolism.

Several important clinical implications can be drawn from our findings. First, CTI is calculated from laboratory parameters (hsCRP, triglycerides, and FBG) that are readily accessible and cost‐effective, making it a practical and scalable tool for risk stratification. Identifying ACS patients with prior CABG who have a high CTI may alert clinicians to a subgroup with substantial residual risk despite successful PCI. Second, such identification may facilitate a more targeted therapeutic strategy. For example, patients with elevated CTI may derive greater benefit from intensive lipid‐lowering therapy with proprotein convertase subtilisin/kexin type 9 (PCSK9) inhibitors to achieve extremely low levels of LDL‐C [49, 50]. In addition, these patients may represent an appropriate population for anti‐inflammatory therapies such as colchicine, which has demonstrated significant cardiovascular benefit in recent trials (COLCOT [Colchicine Cardiovascular Outcomes Trial] and LoDoCo2 [Low‐Dose Colchicine 2]) by targeting the NOD‐like receptor protein 3 inflammasome—a pivotal mediator linking metabolic stress to inflammation [51, 52]. CTI could potentially function as a biomarker to identify candidates for such therapies. Finally, a high CTI underscores the importance of lifestyle modifications, such as adherence to a Mediterranean diet and regular physical activity, as well as consideration of metformin, which has pleiotropic effects that improve insulin sensitivity and may confer anti‐inflammatory benefits [53]. Newer antidiabetic agents with established cardiovascular benefit, such as glucagon‐like peptide 1 receptor agonists and sodium–glucose cotransporter 2 inhibitors, which also modulate inflammatory pathways [54], may also be considered in these patients irrespective of their diabetes status.

## 5. Study Limitations

Although our study provides robust evidence, there are several limitations that should be taken into account in the interpretation of our findings. First, the single‐center retrospective design carries an inherent risk of residual confounding despite rigorous statistical adjustment for a wide range of clinically relevant variables. Unmeasured or uncontrolled variables, such as physical activity levels, dietary patterns, psychosocial stress, and genetic predisposition, may have influenced both CTI and cardiovascular outcomes. Second, the enrollment period spanned 13 years (2008–2021). Major technological advances in interventional cardiology during this period may have influenced the prognosis of patients undergoing PCI. However, owing to database limitations, we were unable to analyze variables reflecting these technological developments, such as the transition from first‐ to third‐generation drug‐eluting stents, modifications in dual antiplatelet therapy strategies, or the increased use of imaging‐guided PCI. Nevertheless, subgroup analysis stratifying the cohort into two time periods (2008–2015 vs. 2016–2021) demonstrated that the relationship between CTI and MACCE remained consistent across both strata (HR per 1‐unit increase: 1.863 [95% CI: 1.532–2.264] and 1.790 [95% CI: 1.552–2.065], respectively; *p* for interaction = 0.858). Third, the study cohort was composed solely of ACS patients with prior CABG who underwent PCI; therefore, these results cannot necessarily be extrapolated to patients managed medically or to those undergoing alternative revascularization strategies. Fourth, CTI is a novel index, and its formula as well as specific cutoff values for clinical decision‐making require external validation in independent multicenter prospective cohorts of similarly high‐risk patients. Fifth, data on long‐term medication adherence during follow‐up were unavailable, which may have influenced the outcomes. Finally, the mechanisms linking CTI to adverse cardiovascular outcomes are inferred from the established biology of its components; future mechanistic studies incorporating advanced vascular imaging modalities (e.g., optical coherence tomography and intravascular ultrasound) to assess plaque morphology or omics approaches (e.g., proteomics and metabolomics) may provide deeper insights into the specific biological pathways involved.

## 6. Conclusions

In conclusion, CTI serves as a robust, independent, and consistent predictor of long‐term MACCE in ACS patients with prior CABG who underwent PCI. This study identified a CTI value of 9.04 as a threshold associated with a poor prognosis. By quantifying the synergistic burden of systemic inflammation and insulin resistance, CTI provides a more comprehensive representation of the “meta‐inflammatory” phenotype prevalent in this complex, high‐risk population. Integration of CTI into clinical practice may allow for earlier stratification and identification of patients at elevated risk who could benefit from a personalized, multifaceted management strategy targeting both inflammatory and metabolic pathways. It is necessary to carry out future prospective studies to ascertain the validity of CTI and to evaluate its application in guiding targeted therapies.

**Nomenclature**
ACS: Acute coronary syndrome
AMI: Acute myocardial infarction
ANOVA: One‐way analysis of variance
AUC: Area under the curve
BMI: Body mass index
CABG: Coronary artery bypass grafting
CI: Confidence interval
CKD‐EPI: Chronic Kidney Disease Epidemiology Collaboration
cNRI: Continuous net reclassification improvement
CRP: C‐reactive protein
CTI: C‐reactive protein–triglyceride glucose index
eGFR: Estimated glomerular filtration rate
FBG: Fasting blood glucose
GRACE: Global Registry of Acute Coronary Events
HbA1c: Glycosylated hemoglobin A1c
HDL‐C: High‐density lipoprotein cholesterol
HOMA‐IR: Homeostasis model assessment of insulin resistance
HR: Hazard ratio
hsCRP: High‐sensitivity C‐reactive protein
IDI: Integrated discrimination improvement
IL: Interleukin
LDL‐C: Low‐density lipoprotein cholesterol
LMCA: Left main coronary artery
LVEF: Left ventricular ejection fraction
MACCE: Major adverse cardiovascular and cerebrovascular events
MI: Myocardial infarction
NF‐κB: Nuclear factor kappa B
NSTEMI: Non‐ST‐segment elevation myocardial infarction
PCI: Percutaneous coronary intervention
PCSK9: Proprotein convertase subtilisin/kexin type 9
ROC: Receiver operating characteristic
SBP: Systolic blood pressure
SCr: Serum creatinine
STEMI: ST‐segment elevation myocardial infarction
SVG: Saphenous vein graft
TIMI: Thrombolysis in myocardial infarction
TNF‐α: Tumor necrosis factor‐α
TyG: Triglyceride–glucose
UA: Unstable angina.

## Author Contributions

**Xiaoteng Ma**: conceptualization, data curation, methodology, writing – original draft. **Huijun Chu**: conceptualization, methodology, writing – original draft. **Qiuxuan Li and Yuxiu Yang**: data curation. **Zhijian Wang**: supervision, validation, writing – review and editing.

## Funding

This work was supported by the grant from the Youth Program of the National Natural Science Foundation of China (Grant 82200405), General Program of the National Natural Science Foundation of China (Grant 82370449), General Program of the Beijing Municipal Natural Science Foundation (Grant 7232039), the Capital’s Funds for Health Improvement and Research (Grant 2022‐2‐1052), and the Beijing Hospitals Authority Incubating Program (Grant PX2021027).

## Ethics Statement

This study was approved by the institutional review board of the Beijing Anzhen Hospital, Capital Medical University (Number 2025349x). Given the retrospective design of this study, the requirement for informed consent was waived.

## Consent

The authors have nothing to report.

## Conflicts of Interest

The authors declare no conflicts of interest.

## Supporting Information

Additional supporting information can be found online in the Supporting Information section.

## Supporting information


**Supporting Information 1** Table S1. Adverse cardiovascular outcomes according to CTI tertiles during follow‐up.

**Supporting Information 2** Table S2. Multivariable Cox proportional hazards model including CTI as a continuous variable, the GRACE risk score, and other confounders for predicting MACCE.

**Supporting Information 3** Table S3. Multivariable Cox proportional hazards model including CTI as a categorical variable (CTI tertiles), selected components of the GRACE risk score, and other confounders for predicting MACCE.

**Supporting Information 4** Table S4. Multivariable Cox proportional hazards model including CTI as a continuous variable, selected components of the GRACE risk score, and other confounders for predicting MACCE.

## Data Availability

The datasets used during the current study are available from the corresponding author upon reasonable request.
