# Supplementary material for: Association Between C‐Reactive Protein–Triglyceride Glucose Index and Adverse Cardiovascular Outcomes in Acute Coronary Syndrome Patients With Prior Coronary Artery Bypass Grafting
Source: Mediators Inflamm. 2026 Jun 8;2026:7921309. doi: 10.1155/mi/7921309 (PMC13244252; doi:10.1155/mi/7921309)
Supplement: Supplementary file 1 — Supporting Information 1 Table S1. Adverse cardiovascular outcomes according to CTI tertiles during follow‐up. [file MI-2026-7921309-s001.docx]

**Table S1. Adverse cardiovascular outcomes according to CTI tertiles during follow-up**

| Adverse cardiovascular outcomes | All patients  n = 1,195 | Lowest tertile  n = 398 | Middle tertile  n = 399 | Highest tertile  n = 398 | *P* value |
| --- | --- | --- | --- | --- | --- |
| MACCE, n (%) | 366 (30.6) | 46 (11.6) | 137 (34.3) | 183 (46.0) | < 0.001 |
| Key secondary endpoint, n (%) | 178 (14.9) | 21 (5.3) | 65 (16.3) | 92 (23.1) | < 0.001 |
| All-cause death, n (%) | 90 (7.5) | 11 (2.8) | 31 (7.8) | 48 (12.1) | < 0.001 |
| Nonfatal stroke, n (%) | 32 (2.7) | 3 (0.8) | 10 (2.5) | 19 (4.8) | 0.002 |
| Nonfatal MI, n (%) | 56 (4.7) | 7 (1.8) | 24 (6.0) | 25 (6.3) | 0.003 |
| Unplanned revascularization, n (%) | 188 (15.7) | 25 (6.3) | 72 (18.0) | 91 (22.9) | < 0.001 |

The key secondary endpoint was a composite of all-cause death, nonfatal stroke, or nonfatal MI. CTI, C-reactive protein–triglyceride glucose index; MACCE, major adverse cardiovascular and cerebrovascular events; MI, myocardial infarction.
