# Supplementary material for: Association Between C‐Reactive Protein–Triglyceride Glucose Index and Adverse Cardiovascular Outcomes in Acute Coronary Syndrome Patients With Prior Coronary Artery Bypass Grafting
Source: Mediators Inflamm. 2026 Jun 8;2026:7921309. doi: 10.1155/mi/7921309 (PMC13244252; doi:10.1155/mi/7921309)
Supplement: Supplementary file 2 — Supporting Information 2 Table S2. Multivariable Cox proportional hazards model including CTI as a continuous variable, the GRACE risk score, and other confounders for predicting MACCE. [file MI-2026-7921309-s002.docx]

**Table S2. Multivariable Cox proportional hazards model including CTI as a continuous variable, the GRACE risk score, and other confounders for predicting MACCE**

|  | **Univariate analysis** | | **Multivariate analysis** | |
| --- | --- | --- | --- | --- |
| **Variables** | **HR (95% CI)** | **P value** | **HR (95% CI)** | **P value** |
| CTI | 1.799 (1.602-2.019) | <0.001 | 1.801 (1.556-2.085) | <0.001 |
| GRACE risk score | 1.007 (1.002-1.011) | 0.006 | 1.003 (0.998-1.008) | 0.296 |
| Male sex | 0.850 (0.673-1.074) | 0.173 | 0.931 (0.726-1.193) | 0.296 |
| BMI | 1.032 (0.999-1.067) | 0.054 | 1.003 (0.970-1.038) | 0.846 |
| Hypertension | 1.292 (0.995-1.677) | 0.054 | 1.139 (0.871-1.489) | 0.343 |
| Diabetes | 1.173 (0.952-1.446) | 0.134 | 0.846 (0.643-1.112) | 0.230 |
| Renal dysfunction | 1.530 (1.109-2.112) | 0.010 | 1.174 (0.830-1.659) | 0.364 |
| Past PCI | 1.300 (1.045-1.617) | 0.019 | 1.144 (0.882-1.484) | 0.312 |
| Previous stroke | 1.272 (0.938-1.724) | 0.121 | 1.240 (0.907-1.694) | 0.177 |
| Chronic lung disease | 0.651 (0.374-1.134) | 0.130 | 0.644 (0.369-1.123) | 0.121 |
| LDL-C | 1.005 (1.002-1.008) | <0.001 | 1.002 (0.999-1.006) | 0.118 |
| HDL-C | 0.983 (0.971-0.995) | 0.006 | 1.001 (0.988-1.015) | 0.884 |
| HbA1c | 1.101 (1.025-1.183) | 0.009 | 0.977 (0.884-1.079) | 0.648 |
| Years since CABG | 1.034 (1.011-1.057) | 0.003 | 1.022 (0.996-1.049) | 0.091 |
| The index PCI as the first PCI after CABG | 0.706 (0.510-0.979) | 0.037 | 0.843 (0.560-1.270) | 0.414 |
| PCI in native and/or graft vessels |  | 0.037 |  | 0.236 |
| PCI in only native vessels | ref |  | ref |  |
| PCI in only graft vessels | 1.436 (1.064-1.938) | 0.018 | 2.721 (0.375-19.730) | 0.322 |
| PCI in both native and graft vessels | 0.807 (0.480-1.357) | 0.418 | 1.777 (0.228-13.871) | 0.584 |
| Native vessel intervened: LMCA | 0.681 (0.481-0.966) | 0.031 | 0.833 (0.584-1.190) | 0.315 |
| Graft vessel intervened: SVG | 1.215 (0.928-1.591) | 0.157 | 0.395 (0.054-2.907) | 0.361 |
| Target vessel revascularization successful | 0.577 (0.364-0.916) | 0.020 | 0.595 (0.372-0.953) | 0.031 |

HR indicates hazard ratio; 95% CI, 95% confidence interval. Other abbreviations as in Tables 1 and 2.
