# Supplementary material for: Association Between C‐Reactive Protein–Triglyceride Glucose Index and Adverse Cardiovascular Outcomes in Acute Coronary Syndrome Patients With Prior Coronary Artery Bypass Grafting
Source: Mediators Inflamm. 2026 Jun 8;2026:7921309. doi: 10.1155/mi/7921309 (PMC13244252; doi:10.1155/mi/7921309)
Supplement: Supplementary file 3 — Supporting Information 3 Table S3. Multivariable Cox proportional hazards model including CTI as a categorical variable (CTI tertiles), selected components of the GRACE risk score, and other confounders for predicting MACCE. [file MI-2026-7921309-s003.docx]

**Table S3. Multivariable Cox proportional hazards model including CTI as a categorical variable (CTI tertiles), selected components of the GRACE risk score, and other confounders for predicting MACCE**

|  | **Univariate analysis** | | **Multivariate analysis** | |
| --- | --- | --- | --- | --- |
| **Variables** | **HR (95% CI)** | **P value** | **HR (95% CI)** | **P value** |
| CTI tertiles |  | <0.001 |  | <0.001 |
| Lowest tertile | ref |  | ref | ref |
| Middle tertile | 3.173 (2.271-4.432) | <0.001 | 2.849 (2.012-4.033) | <0.001 |
| Highest tertile | 4.308 (3.118-5.953) | <0.001 | 3.949 (2.764-5.643) | <0.001 |
| Age | 1.013 (1.000-1.026) | 0.046 | 1.013 (0.999-1.028) | 0.078 |
| Male sex | 0.850 (0.673-1.074) | 0.173 | 0.956 (0.740-1.234) | 0.727 |
| BMI | 1.032 (0.999-1.067) | 0.054 | 1.004 (0.970-1.039) | 0.822 |
| SBP at admission | 1.008 (1.002-1.014) | 0.008 | 1.007 (1.001-1.014) | 0.031 |
| HR at admission | 1.016 (1.007-1.026) | 0.001 | 1.010 (1.000-1.020) | 0.060 |
| Hypertension | 1.292 (0.995-1.677) | 0.054 | 1.079 (0.821-1.417) | 0.586 |
| Diabetes | 1.173 (0.952-1.446) | 0.134 | 0.814 (0.618-1.071) | 0.141 |
| Renal dysfunction | 1.530 (1.109-2.112) | 0.010 | 1.110 (0.781-1.578) | 0.562 |
| Previous MI | 1.163 (0.946-1.428) | 0.151 | 1.056 (0.849-1.312) | 0.626 |
| Past PCI | 1.300 (1.045-1.617) | 0.019 | 1.213 (0.935-1.574) | 0.146 |
| Previous stroke | 1.272 (0.938-1.724) | 0.121 | 1.278 (0.935-1.747) | 0.123 |
| Chronic lung disease | 0.651 (0.374-1.134) | 0.130 | 0.640 (0.365-1.121) | 0.118 |
| LDL-C | 1.005 (1.002-1.008) | <0.001 | 1.003 (0.999-1.006) | 0.104 |
| HDL-C | 0.983 (0.971-0.995) | 0.006 | 0.995 (0.982-1.009) | 0.506 |
| HbA1c | 1.101 (1.025-1.183) | 0.009 | 1.035 (0.940-1.140) | 0.482 |
| Years since CABG | 1.034 (1.011-1.057) | 0.003 | 1.017 (0.991-1.044) | 0.194 |
| The index PCI as the first PCI after CABG | 0.706 (0.510-0.979) | 0.037 | 0.881 (0.585-1.327) | 0.545 |
| PCI in native and/or graft vessels |  | 0.037 |  | 0.201 |
| PCI in only native vessels | ref |  | ref |  |
| PCI in only graft vessels | 1.436 (1.064-1.938) | 0.018 | 3.290 (0.449-24.090) | 0.241 |
| PCI in both native and graft vessels | 0.807 (0.480-1.357) | 0.418 | 2.159 (0.277-16.851) | 0.463 |
| Native vessel intervened: LMCA | 0.681 (0.481-0.966) | 0.031 | 0.848 (0.594-1.210) | 0.362 |
| Graft vessel intervened: SVG | 1.215 (0.928-1.591) | 0.157 | 0.346 (0.047-2.571) | 0.300 |
| Target vessel revascularization successful | 0.577 (0.364-0.916) | 0.020 | 0.641 (0.400-1.027) | 0.065 |

HR indicates hazard ratio; 95% CI, 95% confidence interval. Other abbreviations as in Tables 1 and 2.
