# Supplementary material for: Association Between C‐Reactive Protein–Triglyceride Glucose Index and Adverse Cardiovascular Outcomes in Acute Coronary Syndrome Patients With Prior Coronary Artery Bypass Grafting
Source: Mediators Inflamm. 2026 Jun 8;2026:7921309. doi: 10.1155/mi/7921309 (PMC13244252; doi:10.1155/mi/7921309)
Supplement: Supplementary file 4 — Supporting Information 4 Table S4. Multivariable Cox proportional hazards model including CTI as a continuous variable, selected components of the GRACE risk score, and other confounders for predicting MACCE. [file MI-2026-7921309-s004.docx]

**Table S4. Multivariable Cox proportional hazards model including CTI as a continuous variable, selected components of the GRACE risk score, and other confounders for predicting MACCE**

|  | **Univariate analysis** | | **Multivariate analysis** | |
| --- | --- | --- | --- | --- |
| **Variables** | **HR (95% CI)** | **P value** | **HR (95% CI)** | **P value** |
| CTI | 1.799 (1.602-2.019) | <0.001 | 1.841 (1.583-2.141) | <0.001 |
| Age | 1.013 (1.000-1.026) | 0.046 | 1.016 (1.001-1.031) | 0.034 |
| Male sex | 0.850 (0.673-1.074) | 0.173 | 0.983 (0.760-1.272) | 0.897 |
| BMI | 1.032 (0.999-1.067) | 0.054 | 1.001 (0.968-1.035) | 0.959 |
| SBP at admission | 1.008 (1.002-1.014) | 0.008 | 1.007 (1.001-1.014) | 0.031 |
| HR at admission | 1.016 (1.007-1.026) | 0.001 | 1.008 (0.998-1.018) | 0.118 |
| Hypertension | 1.292 (0.995-1.677) | 0.054 | 1.066 (0.812-1.399) | 0.646 |
| Diabetes | 1.173 (0.952-1.446) | 0.134 | 0.841 (0.639-1.107) | 0.217 |
| Renal dysfunction | 1.530 (1.109-2.112) | 0.010 | 1.053 (0.740-1.500) | 0.774 |
| Previous MI | 1.163 (0.946-1.428) | 0.151 | 1.104 (0.889-1.371) | 0.372 |
| Past PCI | 1.300 (1.045-1.617) | 0.019 | 1.145 (0.882-1.487) | 0.310 |
| Previous stroke | 1.272 (0.938-1.724) | 0.121 | 1.221 (0.893-1.670) | 0.210 |
| Chronic lung disease | 0.651 (0.374-1.134) | 0.130 | 0.630 (0.360-1.100) | 0.104 |
| LDL-C | 1.005 (1.002-1.008) | <0.001 | 1.002 (0.999-1.005) | 0.187 |
| HDL-C | 0.983 (0.971-0.995) | 0.006 | 0.999 (0.985-1.012) | 0.845 |
| HbA1c | 1.101 (1.025-1.183) | 0.009 | 0.965 (0.872-1.068) | 0.491 |
| Years since CABG | 1.034 (1.011-1.057) | 0.003 | 1.018 (0.992-1.044) | 0.182 |
| The index PCI as the first PCI after CABG | 0.706 (0.510-0.979) | 0.037 | 0.836 (0.554-1.260) | 0.391 |
| PCI in native and/or graft vessels |  | 0.037 |  | 0.299 |
| PCI in only native vessels | ref |  | ref |  |
| PCI in only graft vessels | 1.436 (1.064-1.938) | 0.018 | 2.314 (0.318-16.862) | 0.408 |
| PCI in both native and graft vessels | 0.807 (0.480-1.357) | 0.418 | 1.542 (0.197-12.084) | 0.680 |
| Native vessel intervened: LMCA | 0.681 (0.481-0.966) | 0.031 | 0.836 (0.586-1.194) | 0.325 |
| Graft vessel intervened: SVG | 1.215 (0.928-1.591) | 0.157 | 0.482 (0.065-3.573) | 0.475 |
| Target vessel revascularization successful | 0.577 (0.364-0.916) | 0.020 | 0.613 (0.382-0.983) | 0.042 |

HR indicates hazard ratio; 95% CI, 95% confidence interval. Other abbreviations as in Tables 1 and 2.
